# An analysis of urine and serum amino acids in critically ill patients upon admission by means of targeted LC–MS/MS: a preliminary study

**DOI:** 10.1038/s41598-021-99482-8

**Published:** 2021-10-07

**Authors:** Magdalena Mierzchała-Pasierb, Małgorzata Lipińska-Gediga, Mariusz G. Fleszar, Łukasz Lewandowski, Paweł Serek, Sylwia Płaczkowska, Małgorzata Krzystek-Korpacka

**Affiliations:** 1 Department of Biochemistry and Immunochemistry, Wroclaw Medical University, Chałubinskiego 10, 50-368 Wrocław, Poland; 2 Department of Anesthesiology and Intensive Care, Wroclaw Medical University, Borowska 213, 50-556 Wrocław, Poland; 3 Diagnostics Laboratory for Teaching and Research, Wroclaw Medical University, Borowska 211A, 50-556 Wrocław, Poland

**Keywords:** Medical research, Biomarkers, Biomarkers, Diagnostic markers, Infectious diseases, Metabolic disorders

## Abstract

Sepsis, defined as a dysregulated host response to infection, causes the interruption of homeostasis resulting in metabolic changes. An examination of patient metabolites, such as amino acids, during the early stage of sepsis may facilitate diagnosing and assessing the severity of the sepsis. The aim of this study was to compare patterns of urine and serum amino acids relative to sepsis, septic shock and survival. Urine and serum samples were obtained from healthy volunteers (n = 15) once or patients (n = 15) within 24 h of a diagnosis of sepsis or septic shock. Concentrations of 25 amino acids were measured in urine and serum samples with liquid chromatography-electrospray mass spectrometry. On admission in the whole cohort, AAA, ABA, mHis, APA, Gly-Pro and tPro concentrations were significantly lower in the serum than in the urine and Arg, Gly, His, hPro, Leu, Ile, Lys, Orn, Phe, Sarc, Thr, Tyr, Asn and Gln were significantly higher in the serum than in the urine. The urine Gly-Pro concentration was significantly higher in septic shock than in sepsis. The serum Cit concentration was significantly lower in septic shock than in sepsis. The urine ABA, mHis and Gly-Pro, and serum Arg, hPro and Orn concentrations were over two-fold higher in the septic group compared to the control group. Urine and serum amino acids measured in septic patients on admission to the ICU may shed light on a patient’s metabolic condition during sepsis or septic shock.

## Introduction

Sepsis, defined as a dysregulated host response to infection causing life-threatening organ dysfunction, is characterized by a relatively high mortality rate, so prompt diagnosis and early initiation of the appropriate therapy are critical. Finding biomarkers, including metabolic ones, may increase the understanding of the pathogenesis and pathophysiology of sepsis and may have prognostic benefit in septic patient treatment and outcome^1,2^.

Current sepsis investigations are focused on biomarkers as indicies of the severity of sepsis or septic shock at an early stage. More than 200 sepsis biomarkers have been identified and evaluated. It has been suggested that sepsis could be diagnosed by detecting increased concentrations of the parameters that are commonly assessed in clinical practice, such as procalcitonin (PCT)^3^, C-reactive protein (CRP)^4^, lipopolisaccharide-binding protein (LBP)^5^, and pro-atrial natriuretic peptide (pro-ANP)^6^. However, they are insufficiently sensitive or specific as standard tools for the early diagnosis of sepsis^4^. Therefore, the determination of new markers or multi-marker panels remains a priority for increasing the survival rates of ICU patients^7–9^.

The study of metabolome (metabolomics) is gaining increasing attention and is believed to have the potential to alter clinical practice in sepsis^10,11^. In fact, it is believed that putting too much pressure on sepsis as an inflammatory condition and not addressing other contributing factors, including profound changes in metabolic homeostasis, might be the reason for the failure of many biomarkers and clinical trials^12^. In sepsis or septic shock circulating cytokines and catecholamines cause metabolic changes, but the excessive focus on assessing sepsis and septic shock only as a inflammatory condition and ignoring profound changes in metabolic homeostasis resulted in the failure of numerous clinical trials and biomarkers involved in diagnosing and treating septic states^13^.

Among the various metabolites, amino acids have frequently been reported as having been altered in a number of pathological conditions^14^. In humans, skeletal muscles are the main source of peptide-bound and free amino acids, but the major site of extensive catabolism is in the small intestine^15^. Amino acids fulfill a wide range of functions as glucogenic substrates, nitrogen carriers, and the precursors of signal transducers, nucleotides and neurotransmitters. They regulate protein turnover, enzyme activity and ion fluxes and affect cell signaling and inflammatory reactions by participating in the regulation of gene expression and protein phosphorylation. They are also responsible for maintaining organ and body protein homeostasis, which is severely disturbed in the course of sepsis and includes not only the immune-inflammatory aspect but also the massively disrupted metabolic homeostasis associated with an overall catabolic state^16^. The metabolic changes occurring in sepsis represent both systemic and cellular alterations and result in aberrations in the entire metabolic response^17,18^.

Many contemporary studies focus on the measurement of amino acids as potentially prognostic and diagnostic markers in the context of various physiological and pathological conditions. Moreover, changes in the profile of amino acids may also reflect the status of different diseases^19^. One such pathological condition for which the determination of amino acids may have potential value is sepsis. Recently, there has been an increase in the number of scientific papers which have studied the relevance of amino acid concentrations in sepsis or septic shock^7,11,20^.

Our previous studies were focused on measuring the concentrations of amino acids in the serum of septic patients^21^. The obtained results encouraged us to perform further experiments on the associations of amino acids.

Hence, the aim of the present study was to i) determine the urine and serum concentrations of selected amino acids with the use of targeted liquid chromatography coupled with tandem mass spectrometry (LC–MS/MS), ii) identify patterns in the urine and serum in sepsis and septic shock among survivor and non-survivor subgroups, iii) examine the possible correlation of amino acids with the Acute Physiology and Chronic Health Evaluation (APACHE II) and Sequential Organ Failure Assessment (SOFA) scores and biochemical and hematological parameters.

## Results

### Differences in amino acids in the urine and serum in the control group and septic patients

The comparison of the concentrations of amino acids in the urine and serum (expressed as µmol/L) between the control group and septic patients are presented in Table 1. In septic patients, urine AAA, His, mHis, APA, and tPro and serum AAA, Gly-Pro, tPro were significantly lower than in the control group. Serum Arg, ABA, Gly, His, hPro, Leu, Ile, Lys, mHis, Orn, Phe, Sarc, Thr, Tyr, Asn, Gln, and APA were significantly higher than in the control group.

**Table 1:** A comparison of the amino acids in the urine and serum in the control group and septic patients.

Variable	Urine			Serum		
	Control (n = 15)	Septic patients (n = 15)	*p*	Control (n = 15)	Septic patients (n = 15)	*p*
Aminoadipic acid (AAA)	{29.60; **31.70**; 58.25}	{23.65; **25.70**; 29.25}	**0.0007**	{27.21; **29.09**; 31.38}	{22.55; **22.80**; 26.15}	**0.0164**
Arginine (Arg)	{17.70; **20.40**; 31.15}	{22.60; **29.55**; 61.10}	**0.0145**	{29.10; **88.10**; 105.35}	{124.85; **200.45**; 230.20}	** < 0.0001**
a-Aminoisobutyric acid (ABA)	{21.25; **27.55**; 71.05}	{23.35; **46.65**; 116.20}	0.1607	{21.24; **21.63**; 22.51}	{22.60; **24.55**; 36.10}	**0.0001**
Glycine (Gly)	{111.80; **123.80**; 143.95}	{200.40; **254.65**; 355.65}	** < 0.0001**	{154.75; **601.00**; 709.95}	{733.55; **1192.25**; 1438.80}	**0.0004**
Histidine (His)	{185.50; **236.25**; 358.15}	{41.05; **73.60**; 367.25}	**0.0295**	{27.35; **124.65**; 152.65}	{151.20; **217.60**; 315.45}	**0.0007**
4-Hydroxyproline (hPro)	{12.99; **13.20**; 18.83}	{13.68; **17.64**; 18.31}	0.2328	{16.89; **18.99**; 33.19}	{33.61; **53.29**; 96.99}	** < 0.0001**
Leucine (Leu)	{24.70; **26.15**; 31.00}	{24.85; **29.32**; 43.90}	0.3245	{53.35; **110.80**; 152.55}	{151.75; **241.55**; 325.95}	**0.0001**
Isoleucine (Ile)	{22.40; **23.80**; 33.65}	{21.26; **23.98**; 48.77}	1.0000	{36.95; **66.05**; 89.35}	{114.65; **160.55**; 240.70}	** < 0.0001**
Lysine (Lys)	{44.65; **56.35**; 85.85}	{30.60; **45.10**; 155.55}	0.3892	{95.00; **217.75**; 256.35}	{269.50; **362.60**; 486.10}	**0.0003**
3-Methyl-histidine (mHis)	{117.10; **225.15**; 559.50}	{39.20; **59.10**; 89.85}	**0.0001**	{18.80; **20.20**; 23.45}	{26.05; **30.15**; 44.40}	**0.0010**
Ornithine (Orn)	{25.80; **28.15**; 34.40}	{26.00; **35.75**; 58.10}	0.1873	{39.50; **87.60**; 113.35}	{118.05; **184.75**; 247.85}	**0.0007**
Phenylalanine (Phe)	{32.15; **35.35**; 49.70}	{27.45; **35.15**; 72.10}	0.7130	{37.90; **84.85**; 103.95}	{160.10; **228.05**; 266.60}	** < 0.0001**
Sarcosine (Sarc)	{82.49; **142.18**; 241.64}	{53.74; **130.45**; 314.69}	0.5949	{229.91; **544.65**; 759.26}	{579.10; **855.15**; 1189.31}	**0.0128**
Serine (Ser)	{148.35; **177.10**; 209.50}	{60.95; **135.55**; 259.30}	0.2671	{60.20; **174.15**; 219.60}	{143.25; **205.20**; 336.05}	0.0555
Threonine (Thr)	{42.55; **49.10**; 79.90}	{37.30; **48.40**; 173.95}	1.0000	{64.35; **178.40**; 207.95}	{157.40; **274.75**; 392.80}	**0.0145**
Tyrosine (Tyr)	{44.15; **53.45**; 62.90}	{30.30; **36.10**; 78.75}	0.1160	{49.70; **85.50**; 99.05}	{130.85; **146.05**; 212.70}	** < 0.0001**
Citruline (Cit)	{19.05; **19.40**; 20.05}	{19.50; **19.95**; 31.85}	**0.0295**	{23.45; **33.00**; 42.10}	{28.75; **37.15**; 48.70}	0.2671
Asparagine (Asn)	{26.25; **29.10**; 43.70}	{23.95; **28.30**; 45.70}	0.5125	{23.25; **31.00**; 33.10}	{45.85; **54.53**; 63.15}	** < 0.0001**
Glutamine (Gln)	{67.55; **77.50**; 107.90}	{44.60; **57.25**; 112.25}	0.2671	{76.90; **199.65**; 237.40}	{219.80; **365.95**; 444.65}	**0.0007**
Tryptophan (Trp)	{13.55; **14.10**; 16.10}	{11.90; **13.35**; 17.00}	0.1607	{14.70; **19.90**; 22.15}	{15.70; **19.55**; 21.85}	1.0000
Aminopimelic acid (APA)	{38.97; **38.98**; 39.01}	{38.35; **38.38**; 38.41}	** < 0.0001**	{19.60; **19.60**; 19.70}	{19.60; **19.70**; 19.70}	**0.0408**
Glycine-proline (dipeptide) (Gly-Pro)	{38.07; **38.57**; 40.25}	{37.76; **38.61**; 39.55}	1.0000	{36.87; **36.98**; 37.06}	{19.10; **19.15**; 19.30}	** < 0.0001**
Thioproline (tPro)	{37.70; **37.75**; 37.82}	{37.60; **37.61**; 37.65}	**0.0001**	{37.59; **37.59**; 37.65}	{19.95; **20.00**; 20.10}	** < 0.0001**

Data is presented as {1st quartile; median value; 3rd quartile}; cases in which *p* < 0.05 (U Mann–Whitney test) are marked in bold. The median value is expressed as µmol/L.

### Differences in amino acids in the urine and serum in septic shock or survivor patients

In septic shock, the urine Gly-Pro was significantly higher than in patients with sepsis, but the serum Cit was significantly lower in septic shock. Descriptive statistics are shown in Table 2, where the median value of the amino acid concetration is expressed as µmol/L.

**Table 2:** A comparison of the amino acids in the urine and serum in sepsis and septic shock.

Variable	Urine			Serum		
	Sepsis (n = 6)	Septic shock (n = 9)	*p*	Sepsis (n = 6)	Septic shock (n = 9)	*p*
Aminoadipic acid (AAA)	{24.10; **25.18**; 27.25}	{23.65; **27.50**; 29.90}	0.6070	{22.60; **22.73**; 23.15}	{22.15; **23.55**; 26.15}	0.7756
Arginine (Arg)	{20.30; **25.90**; 29.55}	{24.70; **34.40**; 61.10}	0.3277	{219.35; **228.48**; 247.85}	{121.55; **187.50**; 200.45}	0.0663
a-Aminoisobutyric acid (ABA)	{22.75; **31.25**; 68.15}	{27.90; **72.30**; 116.20}	0.2721	{22.30; **24.88**; 31.90}	{23.65; **24.55**; 36.10}	0.6070
Glycine (Gly)	{216.40; **254.23**; 305.50}	{200.40; **254.65**; 355.65}	0.9546	{780.00; **992.25**; 1438.80}	{701.60; **1192.25**; 1288.75}	0.8639
Histidine (His)	{58.05; **67.15**; 171.75}	{41.05; **102.80**; 367.25}	0.7756	{151.20; **181.55**; 298.20}	{158.55; **261.85**; 315.45}	0.6889
4-Hydroxyproline (hPro)	{13.16; **14.84**; 17.64}	{14.92; **17.80**; 18.31}	0.2721	{36.99; **46.21**; 56.37}	{33.61; **73.85**; 110.64}	0.4559
Leucine (Leu)	{27.30; **28.78**; 35.25}	{24.85; **29.32**; 43.90}	1.0000	{139.25; **202.85**; 368.40}	{198.25; **241.55**; 317.50}	0.7756
Isoleucine (Ile)	{21.26; **23.50**; 31.82}	{21.49; **24.33**; 48.77}	0.6889	{114.65; **159.70**; 240.70}	{129.20; **160.55**; 198.15}	0.9546
Lysine (Lys)	{35.00; **40.48**; 250.85}	{30.60; **60.15**; 148.90}	0.8639	{340.55; **431.23**; 488.00}	{269.50; **349.10**; 377.20}	0.3884
3-Methyl-histidine (mHis)	{39.20; **49.90**; 64.70}	{48.85; **66.90**; 182.20}	0.3277	{28.75; **29.75**; 30.25}	{25.75; **31.55**; 46.20}	0.8639
Ornithine (Orn)	{27.00; **31.73**; 105.35}	{25.90; **35.95**; 37.60}	0.7756	{242.85; **247.75**; 282.75}	{118.05; **160.00**; 184.75}	0.0879
Phenylalanine (Phe)	{28.15; **35.98**; 44.10}	{25.80; **35.15**; 72.10}	0.8639	{160.10; **222.40**; 266.60}	{162.40; **228.05**; 243.70}	1.0000
Sarcosine (Sarc)	{55.49; **132.63**; 241.49}	{53.74; **95.40**; 314.69}	0.9546	{643.85; **805.95**; 1604.50}	{579.10; **856.55**; 1108.01}	0.8639
Serine (Ser)	{60.95; **127.10**; 259.30}	{63.75; **135.55**; 242.05}	1.0000	{166.95; **239.85**; 401.85}	{143.25; **205.20**; 335.90}	0.6070
Threonine (Thr)	{34.75; **60.18**; 173.95}	{38.05; **45.55**; 101.60}	1.0000	{157.40; **329.40**; 490.90}	{182.55; **202.70**; 386.50}	0.6889
Tyrosine (Tyr)	{31.15; **38.55**; 56.50}	{30.30; **36.10**; 78.75}	0.9546	{131.10; **142.75**; 223.55}	{130.85; **146.05**; 164.90}	0.7756
Citruline (Cit)	{19.80; **20.15**; 33.70}	{19.50; **19.85**; 22.50}	0.5287	{43.00; **46.80**; 60.10}	{28.40; **29.55**; 37.15}	**0.0360**
Asparagine (Asn)	{23.95; **32.23**; 45.70}	{24.15; **26.25**; 29.10}	0.8639	{48.20; **54.55**; 67.95}	{45.85; **54.50**; 56.35}	0.6993
Glutamine (Gln)	{40.15; **73.90**; 108.60}	{48.95; **57.05**; 112.25}	0.8639	{227.05; **342.65**; 424.90}	{219.80; **365.95**; 444.65}	0.8639
Tryptophan (Trp)	{12.30; **13.60**; 17.00}	{11.90; **13.35**; 14.80}	0.6889	{15.20; **21.70**; 24.00}	{16.95; **18.60**; 19.55}	0.4559
Aminopimelic acid (APA)	{38.35; **38.36**; 38.38}	{38.37; **38.39**; 38.43}	0.1135	{19.70; **19.70**; 19.70}	{19.60; **19.70**; 19.70}	0.3277
Glycine-proline (dipeptide) (Gly-Pro)	{37.68; **37.72**; 37.84}	{38.61; **38.69**; 39.55}	**0.0360**	{19.10; **19.13**; 19.15}	{19.13; **19.23**; 19.33}	0.2824
Thioproline (tPro)	{37.61; **37.62**; 37.71}	{37.60; **37.61**; 37.64}	0.4559	{19.95; **19.98**; 20.00}	{19.95; **20.00**; 20.20}	0.3884

Data is presented as {1st quartile; median value; 3rd quartile}; cases in which *p* < 0.05 (U Mann–Whitney test) are marked in bold. The median value is expressed as µmol/L.

In non-survivors, the serum hPro and Gln were significantly higher compared to survivors, as shown in Table 3, where the median value of the amino acid concentration is expressed as µmol/L.

**Table 3:** A comparison of the amino acids in the urine and serum in survivors and non-survivors.

Variable	Urine			Serum		
	Survivors (n = 11)	Non-survivors (n = 4)	*p*	Survivors (n = 11)	Non-survivors (n = 4)	*p*
Aminoadipic acid (AAA)	{23.95; **25.70**; 27.90}	{23.43; **26.78**; 250.35}	0.7531	{22.55; **22.70**; 23.55}	{23.45; **25.85**; 31.13}	0.4894
Arginine (Arg)	{20.30; **28.65**; 34.40}	{30.03; **48.23**; 359.28}	0.1377	{124.85; **200.45**; 230.20}	{122.63; **203.00**; 263.20}	0.9495
a-Aminoisobutyric acid (ABA)	{22.75; **46.65**; 116.20}	{25.78; **54.08**; 577.30}	0.5714	{22.60; **24.00**; 31.90}	{23.88; **30.83**; 42.53}	0.6608
Glycine (Gly)	{190.05; **231.10**; 309.05}	{241.15; **435.08**; 1944.70}	0.2256	{780.00; **1192.25**; 1438.80}	{356.45; **969.73**; 1418.33}	0.4894
Histidine (His)	{37.80; **68.45**; 265.05}	{71.93; **275.43**; 459.43}	0.2256	{151.20; **183.35**; 298.20}	{147.95; **288.65**; 364.88}	0.5714
4-Hydroxyproline (hPro)	{13.16; **15.32**; 18.31}	{15.74; **17.84**; 19.30}	0.4894	{32.25; **39.12**; 56.37}	{92.25; **133.06**; 167.73}	**0.0029**
Leucine (Leu)	{24.65; **28.70**; 35.25}	{27.08; **104.31**; 1115.85}	0.3429	{149.50; **198.25**; 368.40}	{242.63; **280.60**; 321.73}	0.3429
Isoleucine (Ile)	{21.26; **23.01**; 31.82}	{34.51; **83.07**; 309.72}	0.2256	{113.20; **150.95**; 240.70}	{176.70; **195.50**; 221.85}	0.2256
Lysine (Lys)	{30.60; **35.85**; 148.90}	{44.80; **107.85**; 735.23}	0.4117	{269.50; **362.60**; 486.10}	{260.43; **416.88**; 487.98}	0.8513
3-Methyl-histidine (mHis)	{38.55; **59.10**; 89.85}	{49.18; **58.20**; 133.65}	0.5714	{26.05; **29.35**; 37.75}	{25.60; **37.98**; 46.48}	0.5714
Ornithine (Orn)	{27.00; **35.75**; 58.10}	{24.83; **31.75**; 62.23}	0.7531	{112.40; **184.75**; 247.85}	{144.70; **191.10**; 232.40}	0.8513
Phenylalanine (Phe)	{27.45; **33.80**; 44.10}	{29.58; **61.98**; 783.35}	0.5714	{160.10; **228.05**; 266.40}	{151.15; **268.45**; 391.73}	0.5714
Sarcosine (Sarc)	{54.66; **130.45**; 241.49}	{45.76; **626.97**; 2190.96}	0.8513	{462.33; **814.47**; 1302.42}	{717.13; **981.58**; 1148.66}	0.7531
Serine (Ser)	{60.95; **89.10**; 242.05}	{97.85; **488.10**; 1131.75}	0.4117	{135.90; **168.80**; 310.90}	{239.58; **335.98**; 338.55}	0.3429
Threonine (Thr)	{34.75; **48.40**; 101.60}	{42.20; **745.53**; 1446.18}	0.2256	{143.85; **202.70**; 392.80}	{262.00; **363.98**; 421.40}	0.4894
Tyrosine (Tyr)	{30.05; **36.10**; 56.50}	{31.38; **55.60**; 423.83}	0.5714	{130.85; **138.50**; 212.70}	{113.70; **151.88**; 233.25}	0.8513
Citruline (Cit)	{19.80; **19.95**; 22.50}	{19.28; **25.68**; 31.85}	0.9495	{29.55; **43.00**; 60.10}	{26.20; **32.78**; 40.38}	0.2256
Asparagine (Asn)	{23.95; **28.30**; 35.70}	{23.28; **91.60**; 214.35}	0.5714	{36.65; **51.35**; 55.30}	{54.05; **59.75**; 78.10}	0.1419
Glutamine (Gln)	{44.60; **57.25**; 108.60}	{46.73; **253.15**; 834.65}	0.5714	{202.00; **274.75**; 424.90}	{405.30; **466.93**; 557.18}	**0.0396**
Tryptophan (Trp)	{11.90; **13.10**; 14.80}	{12.53; **16.68**; 33.35}	0.4894	{15.20; **18.60**; 21.85}	{17.63; **19.70**; 22.68}	0.5714
Aminopimelic acid (APA)	{38.35; **38.37**; 38.41}	{38.38; **38.39**; 38.41}	0.3429	{19.70; **19.70**; 19.70}	{19.60; **19.65**; 20.10}	0.6608
Glycine-proline (dipeptide) (Gly-Pro)	{37.69; **37.84**; 39.55}	{38.61; **39.07**; 46.16}	0.1773	{19.10; **19.15**; 19.30}	{19.10; **19.23**; 19.30}	0.7333
Thioproline (tPro)	{37.60; **37.61**; 37.65}	{37.62; **37.64**; 37.89}	0.4117	{19.90; **20.00**; 20.05}	{19.98; **20.13**; 20.33}	0.1773

Data is presented as {1st quartile; median value; 3rd quartile}; cases in which *p* < 0.05 (U Mann–Whitney test) are marked in bold. The median value is expressed as µmol/L.

### Correlations between the concentration of amino acids and selected clinical parameters in septic patients

On admission, significant correlations were observed between amino acids and clinical parameters. Urine Gly and Trp were moderately, positively correlated with SOFA (ρ = 0.5229 and 0.6271, respectively; *p* = 0.0455 and 0.0124, respectively). Urine His and Asn were moderately, negatively correlated with urea (ρ = −0.5250 and − 0.5893, respectively; *p* = 0.0445 and 0.0208, respectively). Moreover, urine Gly and Sarc were moderately, positively correlated with PCT (ρ = 0.5516 and ρ = 0.5473, respectively; *p* = 0.0409 and 0.0428, respectively). Additionally, serum ABA, Ile, Thr and Trp were moderately, positively correlated with WBC (ρ = 0.65; 0.5321; 0.5143; 0.622, respectively; *p* = 0.0094; 0.0412; 0.0498; 0.0133, respectively).

### Different patterns of amino acid concentrations in the context of urine or serum specimens

**Figure 1:**
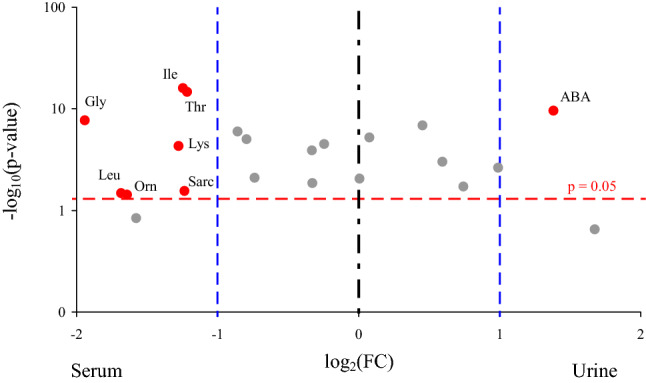
Volcano plot indicating the amino acids with the most meaningful changes analyzed in the serum and urine of the control group based on univariate analysis—y-axis: Mann–Whitney *p* value, x-axis: fold change (FC). Each dot represents one amino acid. The amino acids with significance *p* < 0.05 are located above the red horizontal line. The amino acids with more than a twofold change of a concentration are located on right and left from the respective blue right and left vertical lines. Only red dots indicate amino acids with the clinical and statistical differences. ABA, α-aminoisobutyric acid; Gly, glycine; Ile, isoleucine; Leu, leucine; Lys, lysine; Orn, ornithine; Sarc, sarcosine; Thr, threonine; *p*, significant value.

To reveal the most meaningful differences in the patterns of the urine and serum amino acid concentrations, a volcano plot was performed for both the control group and septic patients. In the control group, the ABA concentration was higher and Gly, Ile, Leu, Lys, Orn, Sarc and Thr concentrations were lower in the urine compared to the serum samples (Fig. 1).

**Figure 2:**
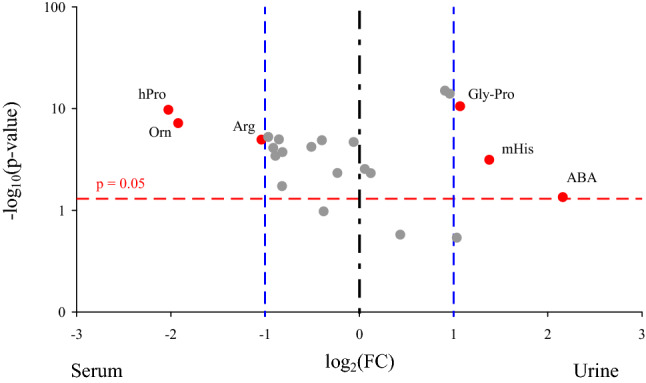
Volcano plot indicating amino acids with the most meaningful changes analyzed in the urine and serum of septic patients on admission based on univariate analysis—y-axis: Mann–Whitney *p* value, x-axis: fold change (FC). Each dot represents one amino acid. The amino acids with significance *p* < 0.05 are located above the red horizontal line. The amino acids with more than a twofold change of a concentration are located on right and left from the respective blue right and left vertical lines. Only red dots indicate amino acids with the clinical and statistical differences. Arg, arginine; ABA, α-aminoisobutyric acid; Gly-Pro, glycine-proline; hPro, 4 hydroxyproline; mHis, 3-methyl-histidine; Orn, ornithine; *p*, significant value.

In septic patients, ABA, mHis and Gly-Pro concentrations were over twofold higher and Arg, hPro and Orn concentrations were over twofold lower in the urine compared to the serum samples (Fig. 2).

**Figure 3:**
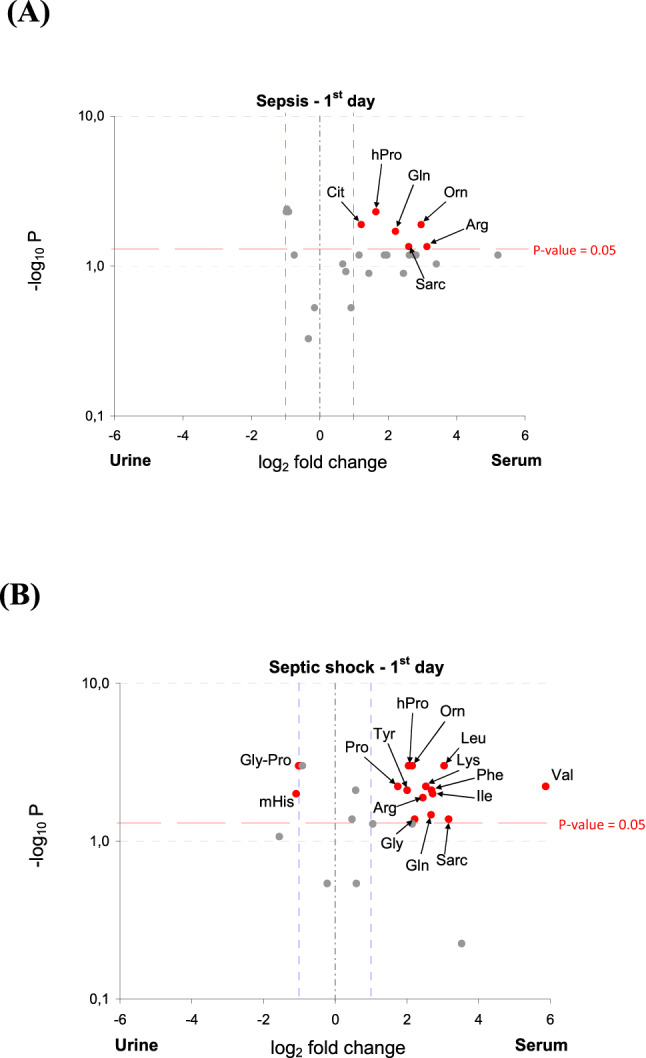
Volcano plot indicating amino acids with the most meaningful changes analyzed in the urine and serum of sepsis (**A**) and septic shock (**B**) on admission based on univariate analysis—y-axis: Mann–Whitney *p* value, x-axis: fold change (FC). Each dot represents one amino acid. The amino acids with significance *p* < 0.05 are located above the red horizontal line. The amino acids with more than a twofold change of a concentration are located on right and left from the respective blue right and left vertical lines. Only red dots indicate amino acids with the clinical and statistical differences. Arg, arginine; Cit, citrulline; Gln, glutamine; Gly, glycine; Gly-Pro, glycine-proline; hPro, 4 hydroxyproline; Ile, isoleucine; Leu, leucine; Lys, lysine; mHis, 3-methyl-histidine; Orn, ornithine; Phe, phenylalanine; Pro, proline; Sarc, sarcosine; Tyr, tyrosine; Val, valine; *p*, significant value.

The amino acid concentrations in septic patients were analyzed relative to the incidence of septic shock and survival. In sepsis, Arg, Cit, Gln, hPro, Orn and Sarc concentrations were over twofold higher in the serum samples (Fig. 3A) compared to the urine. However, in septic shock, the Gly-Pro and mHis concentrations were higher and Arg, Gln, Gly, Ile, hPro, Leu, Lys, Orn, Phe, Pro, Sarc, Tyr, Val concentrations were lower in the urine (Fig. 3B).

**Figure 4:**
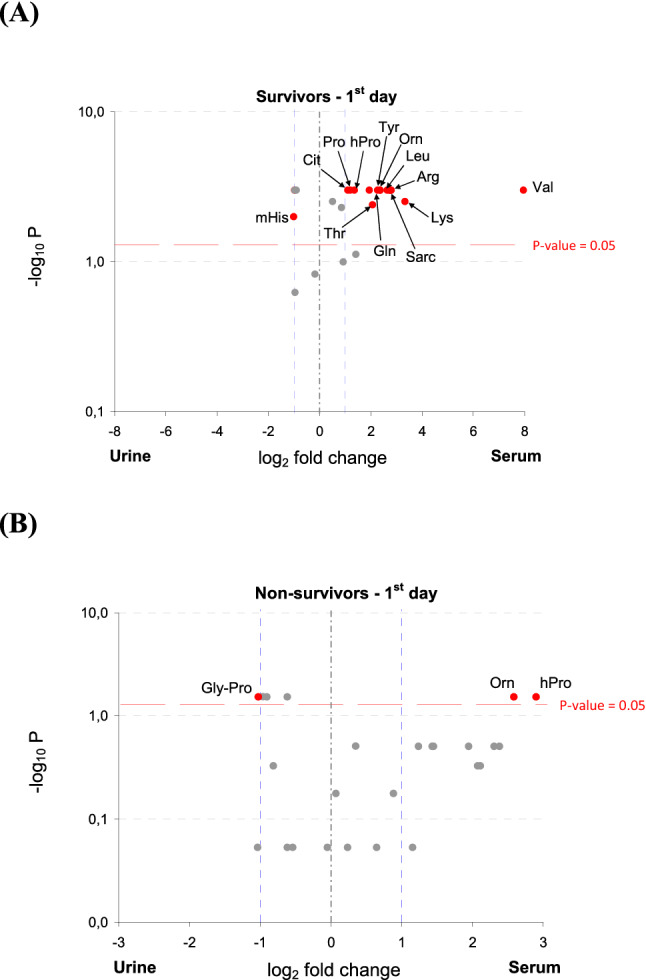
Volcano plot indicating the amino acids with the most meaningful changes analyzed in the urine and serum of survivors (**A**) and non-survivors (**B**) on admission based on univariate analysis—y-axis: Mann–Whitney *p* value, x-axis: fold change (FC). Each dot represents one amino acid. The amino acids with significance *p* < 0.05 are located above the red horizontal line. The amino acids with more than a twofold change of a concentration are located on right and left from the respective blue right and left vertical lines. Only red dots indicate amino acids with the clinical and statistical differences. Arg, arginine; Cit, citrulline; Gln, glutamine; Gly-Pro, glycine-proline; hPro, 4-hydroxyproline; Leu, leucine; Lys, lysine; mHis, 3-methyl-histidine; Orn, ornithine; Pro, proline; Sarc, sarcosine; Thr, threonine; Tyr, tyrosine; *p*, significant value.

In survivors, the concentration of mHis was over twofold higher and Cit, Pro, hPro, Tyr, Orn, Leu, Arg, Lys, Sarc, Gln, Thr and Val concentrations were lower in the urine compared to the serum samples (Fig. 4A). In non-survivors, the Gly-Pro concentration was higher and hPro and Orn concentrations were lower in the urine (Fig. 4B).

### Survival analysis

The saturated model included the following continuous variables APACHE II score (1st day), SOFA score (1st day), age, serum hPro and Gln concentration; and the following nominal variables: the occurrence of septic shock and sex.

Both the stepwise regression and backward elimination models of the best fit are shown in Table 4. According to the backward elimination model, a one-unit change in the values of the SOFA score or the Gln concentration in the serum obtained on the first day are associated with a change in the risk of mortality of approximately 74.17% (*p* = 0.0101) and 1.39% (*p* = 0.0398), respectively. Patients without septic shock had a substantially lower risk of mortality by approximately 98.95% (*p* = 0.0208). Stepwise regression resulted in obtaining a model utilizing two variables: the SOFA score on the first day and the serum Gln concentration. A one-unit change in the SOFA score or Gln concentration in the serum was associated with a change in the risk of mortality of approximately 39.84% (*p* = 0.0069) and 0.83% (*p* = 0.0356), respectively. Since the values of both the AIC and SBC were notably lower (25.04 vs. 29.98 and 27.03 vs. 30.77, respectively) in the model obtained in the process of backward elimination compared to the model obtained with the use of stepwise regression, the former model is a better fit to the dataset used in this study.

**Table 4:** Cox proportional hazard models obtained by means of stepwise regression or backward elimination (maximum of 50 iterations).

Stepwise regression model									
− 2LogL			AIC			SBC		R^2^	
25.98			29.98			30.77		0.77	
Variable	Coefficient	SE	χ^2^	*p*	− 95% CI	95% CI	HR	− 95% CI HR	95% CI HR
SOFA (1)	− 0.5083	0.1883	7.2882	**0.0069**	− 0.8773	− 0.1393	**0.6015**	0.4159	0.8700
Glutamine (Gln)	− 0.0083	0.0040	4.4167	**0.0356**	− 0.0161	− 0.0006	**0.9917**	0.9840	0.9994

Backward elimination model									
− 2LogL			AIC			SBC		R^2^	
15.04			25.04			27.03		0.91	
Variable	Coefficient	SE	χ^2^	*p*	− 95% CI	95% CI	HR	− 95% CI HR	95% CI HR
APACHE II	0.1977	0.1160	2.9022	0.0885	− 0.0298	0.4251	1.2186	0.9707	1.5297
SOFA	− 1.3534	0.5261	6.6181	**0.0101**	− 2.3846	− 0.3223	**0.2583**	0.0921	0.7245
Glutamine (Gln)	− 0.0140	0.0068	4.2281	**0.0398**	− 0.0273	− 0.0007	**0.9861**	0.9731	0.9993
Septic shock: NO	− 2.2801	0.9862	5.3452	**0.0208**	− 4.2130	− 0.3472	**0.0105**	0.0002	0.4994
Sex: MALE	1.7225	0.9765	3.1112	0.0778	− 0.1915	3.6364	31.3410	0.6818	1440.6253

P-values < 0.05 and their corresponding hazard ratios (HR) are marked in bold. AIC, Akaike information criterion; SBC, Schwarz bayesian criterion; SE, standard error; HR, hazard ratio; CI, confidence interval, SOFA, sequential organ failure assessment; Gln, glutamine.

### ROC analysis on the concentration of selected amino acids relative to the incidence of septic shock

Two amino acids were selected for ROC analysis as their concentrations were shown to be significantly different between septic patients with and without septic shock. It is important to note that the use of amino acid concentrations to predict states such as septic shock is a theoretical examination which requires further analysis on larger sample populations.

According to the data set used in this study, the hPro concentration yielded very good predictive power (AUC 0.977 ± 0.035), whereas the Gln concentration was characterized by lower predictive power and a high standard error (AUC 0.864 ± 0.101). Therefore, hPro would be the preferred selection to further study predictive power. According to the Youden index, the hPro concentration of 73.85 µmol/L should be used as a cut-off value to maximize both sensitivity and specificity (1.000 and 0.909, respectively). A cut-off of 110.64 µmol/L would maximize specificity (1.000) at the cost of sensitivity (0.750). The most optimal cut-off value of the Gln concentration is 444.65 µmol/L (sensitivity: 0.750; specificity: 0.909). Lowering the cut-off value to 365.95 µmol/L would maximize sensitivity (1.000) at the cost of rather poor specificity (0.636). On the other hand, a cut-off value of 625.15 µmol/L would yield erroneous sensitivity (0.250), while allowing maximum specificity (1.000).

## Discussion

Measuring amino acids may supply information about the pathomechanism of diseases. It is important to note that some amino acids, such as His play an important role in immunomodulation and influence total antioxidative capacity^22^. Gln plays a crucial role as a non-toxic carrier of nitrogen between organs^23^. During sepsis, Gln is an essential amino acid, due to its an increasing demand for it in relation to the rate of endogenous synthesis^24^. Plasma Cit concentration is determined primarily by the balance between gut Cit synthesis and kidney Cit degradation^25^. Phe and Tyr are substrates for protein synthesis and precursors of physiologically important metabolites, such as catecholamines and thyroid hormones. Increased serum concentrations of these amino acids are associated with rapid protein catabolism, further augmented by sepsis, rather than reflecting the state of decreased hepatic conversion of Phe to Tyr. Phe can be converted into fumarate and this conversion, though being detrimental to the outcome, might act as a supplementary energy supply in anoxic conditions^26^. Arg is reported to have immunotrophic effects and among all the amino acids has the strongest insulinogenic activity, as it stimulates the release of pituitary growth hormone, prolactine and glucagon^23^. Two enzyme families are involved in the main catabolism of Arg, namely the nitric oxide synthases (NOSs), which include inducible NOS (iNOS), neural NOS (nNOS) or endothelial NOS (eNOS), and the arginases^27^. Arg is an intercellular substrate for nitric oxide production in macrophages, improving bactericidal activity and T-cell proliferation and maturation^28^. Moreover, nitric oxide regulates the processes involved in the progression of sepsis, including vascular function and host defence against pathogens^27^.

We decided to assess the metabolites in both the blood and urine, as they represent the current and final metabolic condition during sepsis or septic shock, respectively. To exclude the impact of the response to the introduced therapy, measurements were carried out upon admission to the ICU.

In this study, urine concentrations of Arg, Gly, Cit were found to be significantly higher and APA, AAA, tPro, mHis, His concentrations were significantly lower in septic patients compared to the control group. Serum concentrations of all the analyzed amino acids, except for tPro and Gly-Pro, were significantly higher in septic patients. In this study, the concentration of tPro, which is widely described as an immunomodulator that promotes the survival of leukocytes and phagocytic properties of macrophages^29^, was significantly lower in septic patients than in the control group, probably as a result of an increased demand for it.

In this study, contrary to the findings of Jaurila et al.^30^, the serum His concentration was higher in septic patients compared to the control group. In addition, this study found that the urine His concentration was lower in septic patients. Moreover, the urine and serum concentrations of Gly were significantly higher in septic patients compared to the control groups both in the present study and in the study by Jaurila et al.^30^. Corroborating our findings, the serum His and Phe concentrations were elevated in sepsis also in a pediatric cohort as demonstrated by Mickiewicz et al.^31^. Perhaps, these changes are associated with increased muscle protein turnover, oxidation, decreased energy supply and organ dysfunction in septic patients^31^.

According to Piton et al.^32^, two factors may increase the plasma Cit concentration in critically ill patients: the iNOS, which increases the synthesis of extra-intestinal Cit from Arg, and acute renal failure, a frequent condition in critically ill patients which may cause a decrease in Arg synthesis from Cit in the kidneys leading to an abnormally high plasma Cit concentration^32^. Moreover, as observed by Wijnands et al.^33^, both Arg and Cit deficiencies are linked to poor outcome in septic shock. In our study, the difference in the Cit concentration between septic patients and the control group was insignificant. This finding is not supported by the literature, as Kao et al.^34^ found higher plasma Cit in the control group with balanced metabolism, compared to septic patients. Interestingly the serum Cit concentration differed among septic patients relative to the incidence of septic shock, whereby under conditions of better metabolism, the serum Cit concentration was higher than for patients in a state of septic shock, possibly due to the less disturbed gut synthesis of Cit.

Piton et al.^32^ found that the incidence of septic shock was associated with a lower Cit concentration. Moreover, a low serum Cit concentration was an independent predictor of mortality. Our results are in line with this observation, even 24 h earlier (on admission), although this was statistically insignificant probably due to the low number of non-survivors. Moreover, in the study by Piton et al.^32^, similarily to the findings in this study, the serum Cit concentrations were constant among patients without septic shock and decreased rapidly in patients with septic shock. In our study, the serum but not the urine Phe concentration was significantly higher in septic patients compared to the control group. The Phe concentration did not vary for either septic shock or survival.

On admission the concentrations of several serum and urine amino acids were correlated with clinical and biochemical parameters. To our knowledge, the literature does not feature data that takes into consideration sepsis or septic shock. Hypothetically, some of these correlations may have been incidental, resulting from the small size of the whole cohort and/or covariance with parameters which were not featured in our study.

The Cox proportional hazard regression test revealed that the concentration of glutamine upon admission may be a predictor of the risk of mortality. This finding is supported by the literature, as low Gln concentrations upon admission to the ICU have previously been reported to be correlated with mortality^35–37^. In this study, in contrast to the study of Blauuw et al.^38^, only the serum (but not the urine) Gln concentration was significantly higher in non-survivors compared to survivors. Interestingly, the incidence of septic shock was not associated with changes in either the urine or serum concentrations of Gln. Blaauw et al.^38^ stated that both low and high values of the serum Gln concentration are associated with increased mortality, as changes in the Gln concentration may be U-shaped. Likewise, according to Tsujimoto et al.^39^, both lower and higher values of the serum Gln concentration were reported to be associated with a risk of mortality among patients with sepsis. These authors observed two-way (relatively increased or decreased) changes in serum Gln concentrations in both survivor and non-survivor groups and concluded that increased mortality was associated with extreme Gln concentrations, regardless of whether they were high or low. This observation explains the findings presented here. Our results confirm these observations, but due to the small size of the study group we consider them to be preliminary results which require further research.

One of the explanations of the connection between a concentration of Gln and mortality rate might be the fact that Gln metabolism and balance is strictly linked to the function of the liver. The liver dysfunction observed in sepsis or septic shock (without former failure) may results in the high concentration of serum Gln as a consequence of disturbed Gln metabolism^40^. The Gln is present in high concentration in all organs and tissues and it may account for as much as 50% of amino acids released from whole body protein breakdown^41^. The damage of cell may result in the high concentration of serum Gln, due to the leakage of intracellular Gln, as intracellular Gln concentration exceed its serum concentration^36^. The septic destruction of cells, tissues and organs resulting in the high concentration of serum Gln might be responsible for host death^24^. Though Gln is reffered to a fuel for the immune system, Cruzat et al. stated that for some catabolic patients the changes in plasma Gln concentration, will not necessarily affect and suppress the immune function with no change in mortality risk predictors, such as APACHE II score. According to authors hypoglutaminemia can only be interpreted as an independent variable of mortality and/or poor outcome^40^. Smedberg et al. results confirmed our observation that hyperglutaminemia present at ICU admission was an independent mortality predictor for critically ill patients. Authors concluded that the mechanism behind a serum Gln concentration out of the normal range is likely multifactorial, and still obscure^42^.

Kao et al. reported^24^ that septic patients without the incidence of septic shock showed a higher Arg concentration. The authors linked this fact to decreased de novo Arg synthesis, secondary to decreased Cit production, partially stemming from defects in the conversion of Gln to Cit. In this study, the concentration of Arg was lower in septic shock, although the difference (compared to septic patients with no septic shock) was on the borderline of statistical significance (*p* = 0.0663). Furthermore, in this study, in contrast to another study by Kao et al.^34^, there was no statistically significant difference between survivors and non-survivors in the Arg concentration in the serum or urine. In a study by Garcia-Simon et al.^18^ the urine Gln, Arg and Phe concentrations were lower in non-survivors compared to survivors, in contrast to the results of this study which showed higher serum concentrations of the aforementioned amino acids coupled with higher urine concentrations.

According to the Cox proportional hazard models of the best fit, a one-unit increase in the SOFA score within the first day was associated with a decrease in the risk of mortality. However, it is important to note that, in terms of the risk of mortality, the response or lack of a response to the administered therapy is more of a significant factor than the absolute SOFA score value. This is in line with the observations of Wang et al.^43^ that the SOFA score value did not differ significantly between survivors and non-survivors**.**

In this study there are some limitations which might affect our results. Owing to preliminary character of the research, the sample size was small and our observations require confirmation on a larger cohort. Reference data on amino acid concentration was obtained from volunteers regarded healthy based on self-declared well-being and lack of significant medical history. Moreover, there was difference in urine sample collection, which has been obtained randomly from septic patients within 24 h after ICU admission and diagnosis whereas in case of healthy volunteers in the morning as a second spontaneous urine.

Uncovering differences in the concentrations of amino acids in body fluids is very relevant to immuno-inflammatory diseases, such as sepsis. Although this study should be viewed as preliminary work due to the small sample size, our findings may contribute to the future course of clinical optimization of therapeutic procedures that are based on sets of metabolite concentrations which play a potential role as prognostic parameters.

## Materials and methods

### Patients

This prospective and observational study comprised fifteen adult patients with a diagnosis of sepsis or septic shock who were hospitalized in the Department of Anesthesiology and Intensive Therapy of Wroclaw Medical University, Wroclaw, Poland. The patients were enrolled in a consecutive manner, if they presented with sepsis or septic shock within the first 24 h of admittance into the intensive care unit. The classification of sepsis or septic shock was performed according to the Sepsis-3 criteria^44^. The criteria of exclusion were pregnancy, age below 18 years, a suspicion or confirmation of cancer, treatment with immunosuppressants, ongoing chemotherapy or terminal illness.

All septic patients were treated according to the Surviving Sepsis Campaign guidelines^45^. Two clinical scores such as APACHE II^46^ and SOFA^47^ were calculated upon admission to the ICU and used to assess the clinical status and the extent of multiple organ dysfunction, respectively.

The detailed characteristics of septic patients are presented in Table 5. The most common source of infection was the abdomen (73.3%). The most common system dysfunctions were respiratory (86.7%) and cardiovascular (100%). ICU mortality was 26.7%. Septic patients were followed-up until their discharge from the ICU or death.

**Table 5:** Demographic and clinical data on admission to the ICU.

Parameter	All (n = 15)	Sepsis (n = 6)	Septic shock (n = 9)	Survivors (n = 11)	Non-survivors (n = 4)
Age [years]^*a*^	65.5 (48–84)	67.8 (50–84)	64 (48–82)	65.8 (48–84)	64.7 (56–72)
Sex (female/male)	8/7	3/3	5/4	6/5	2/2
APACHE II^*a*^	21.1 (11–40)	22.8 (16–29)	20 (11–40)	21.2 (11–40)	21 (13–29)
SOFA^*a*^	9.1 (4–17)	10 (5–17)	8.4 (4–12)	8.7 (4–17)	10 (7–12)
**Infection site**^***b***^					
Abdomen	11 (73.3%)	3 (50%)	8 (88.9%)	8 (72.7%)	3 (75%)
Lung	4 (26.7%)	3 (50%)	1 (11.1%)	3 (27.3%)	1 (25%)
**Organ/system failure**^***b***^					
Respiratory	13 (86.7%)	4 (66.7%)	9 (100%)	9 (81.8%)	4 (100%)
Cardiovascular	15 (100%)	6 (100%)	9 (100%)	11 (100%)	4 (100%)
Hepatic	6 (40%)	3 (50%)	3 (33.3%)	4 (36.4%)	2 (50%)
Coagulation	5 (33.3%)	2 (33.3%)	3 (33.3%)	3 (27.3%)	2 (50%)
Renal	8 (53.3%)	4 (66.7%)	4 (44.4%)	6 (54.5%)	2 (50%)
Neurological	8 (53.3%)	4 (66.7%)	4 (44.4%)	6 (54.5%)	2 (50%)
PCT [µg/L]^*a*^	29.4 (1.5–134)	18.7 (2.2–65.6)	35.3 (1.5–134)	23.2 (1.5–70.3)	44.8 (2.2–134)
CRP [mg/L]^*a*^	274.5 (153–535.6)	232.3 (153–354.5)	316.7 (229.8–525.6)	274.5 (153–535.6)	155.2 (53.6–226.1)
WBC [× 10^3^/mm^3^]^*a*^	17.4 (1.8–40.4)	15.5 (7.0–26.8)	18.7 (1.8–40.4)	15.2 (1.8–29.5)	23.3 (7.7–40.4)

Data are expressed as the mean value with range (a) or the actual number of patients (b).

APACHE II, Acute physiology and chronic health evaluation II; SOFA, sequential organ failure assessment; PCT, procalcitonin; CRP, C-reactive protein; WBC, white blood cells count.

The control group comprised fifteen seemingly healthy volunteers who were recruited from the staff of Diagnostics Laboratory for Teaching and Research of the Wroclaw Medical University and consisted of 7 males and 8 females with a mean age 65.2 years. The inclusion criteria were age  > 18, willingness to participate in the study, self-declared well-being, no significant medical history, no known diseases at the moment and not being on any medication while exclusion criteria were pregnancy, hormonal therapy, confirmed co-morbidities like diabetes, cancer or chronic kidney failure or recent surgical interventions.

### Ethical considerations

The study protocol was approved by the Medical Ethics Committee of Wroclaw Medical University and the study was conducted in accordance with the Helsinki Declaration of 1975, as revised in 2008. Informed consent was obtained from both the patients and/or their relatives and from the healthy volunteers.

### Laboratory and clinical parameters

Procalcitonin (PCT), C-reactive protein (CRP), white blood cells counts (WBC), and other laboratory indices were assessed with routine automatic procedures. The clinical data of patients were retrieved from the medical reports.

### Urine and serum sample collection

Urine samples were collected from septic patients as random collection within 24 h after ICU admission and a diagnosis of sepsis or septic shock and from healthy volunteers as morning second spontaneous urine. The urine samples (5 ml) were divided into aliquots and stored at  − 80 °C for amino acid analysis.

Whole blood samples were collected in the morning from the healthy volunteers or within 24 h after ICU admission from the septic patients. Test tubes with a serum-separator were used for whole blood collection. The blood samples were left to clot at room temperature for 15 min and then centrifuged at 720 g for 10 min. The serum was divided into aliquots and stored at  − 80 °C for amino acid analysis.

### Preparation of calibration standards and urine and serum samples

Calibration standards, urine and serum samples were prepared according to the PHENOMENEX EZ:FAAST kit sample preparation procedure (PHENOMENEX EZ:FAAST FREE (PHYSIOLOGICAL) FOR AMINO ACID ANALYSIS BY LC–MS, USA). The preparation of samples and calibration curves was performed exactly as described in the PHENOMENEX EZ:FAAST KIT manual. Due to technical reasons, the chromatographic method was modified and refined in order to obtain optimal separation. Qualification of the equipment was performed by checking the linearity and reproducibility of the calibration curve. It should be highlighted that for diagnostic purposes, the method would need to be fully validated; here we present only a non-clinical application of the method.

The derivatized samples were separated on a NANOACQUITY HSS C18 column (C18-phase, internal diameter 1 mm, length 50 mm, particle size 1.75 μm) at a flow rate of 70 µL/min using a WATERS NANOACQUITY UPLC system (3 μL injection volume) controlled by MASSLYNX software (Waters, USA). The LC eluents were water (A) and acetonitrile (B), both containing 0.1% formic acid. Elution was performed in a linear gradient as follows: 10% B for 3.0 min, from 10 to 40% B for 6.0 min, from 40 to 60% B for 7 min, from 60 to 90% B for 1.0 min, 90% B for 2.0 min and from 90 to 10% B for 0.10 min. Re*-*equilibration time was 2.9 min. This method was adopted from the our previous work^21^.

Mass spectra were recorded in the positive ion mode on a Quadrupole TOF MS equipped with ESI source (XEVO G2 QTOF MS, Waters, USA). Source parameters under the optimized conditions were as follows: 3.0 kV (spray voltage), 30 V (cone voltage), 100 °C (source temperature) and 350 °C (desolvation temperature). Nitrogen was used as the nebulizing (450 L/h) and drying gas (70 L/h). Data acquisition was carried out on MASSLYNX software (Waters, USA) in resolution MS scan mode, with a range of 70–700 m/z (mass-to-charge ratio) and high collision energy ramp from 10 to 25 eV^21^.

Extracted ion chromatograms were used for quantitative analysis of dipeptide Gly-Pro (glycine-proline) and amino acids: His (histidine), Lys (lysine), Leu (leucine), Ile (isoleucine), Phe (phenylalanine), Thr (threonine), Trp (tryptophan), Val (valine), Asn (asparagine), Ser (serine), Arg (arginine), Cit (citrulline), Orn (ornithine), Gln (glutamine), Gly (glycine), Pro (proline), Tyr (tyrosine), AAA (aminoadipic acid), ABA (α-aminoisobutyric acid), APA (aminopimelic acid), mHis (3-methyl-histidine), hPro (4-hydroxyproline), tPro (thioproline), Sarc (sarcosine). The m/z values used are listed in Table 6.

**Table 6:** The retention time and m/z value for amino acid derivatives.

Variable	Retention time	m/z value
Aminoadipic acid (AAA)	15.7600	332.2054
Arginine (Arg)	7.7200	303.2037
a-Aminoisobutyric acid (ABA)	15.0100	333.1814
Glycine (Gly)	10.3800	204.1232
Histidine (His)	13.9900	370.1988
4-Hydroxyproline (hPro)	9.7900	260.1485
Leucine (Leu)	16.0800	260.1848
Isoleucine (Ile)	16.1300	130.0864
Lysine (Lys)	13.0700	361.1872
3-Methyl-histidine (mHis)	7.2600	298.1772
Ornithine (Orn)	13.4800	347.2164
Phenylalanine (Phe)	15.8800	294.1694
Sarcosine (Sarc)	11.6200	218.1393
Serine (Ser)	15.8900	234.1100
Threonine (Thr)	10.1100	248.1886
Tyrosine (Tyr)	17.6900	396.2036
Citruline (Cit)	8.9600	304.1861
Asparagine (Asn)	10.8300	243.1332
Glutamine (Gln)	8.7200	275.1624
Tryptophan (Trp)	15.0100	333.1800
Aminopimelic acid (APA)	17.1400	346.1354
Glycine-proline (dipeptide) (Gly-Pro)	10.4700	301.1760
Thioproline (tPro)	13.8100	262.1129

Data are presented as the actual retention time expressed in minutes and mass-to-charge number of ions, m/z, mass-to-charge ratio.

### Statistical analysis

Statistical analysis was carried out in the STATISTICA 13.3 package, under license of the Wroclaw Medical University, Wroclaw, Poland. All calculated probabilities were two-tailed. The nominal α value for statistical inference was 0.05.

Due to the rather low number of individuals in the study and the presence of outliers or extreme values of the concentrations of selected amino acids in the urine or serum, a non-parametric approach (U Mann–Whitney test) was employed for statistical inference. Spearman’s rho coefficient was used to evaluate possible monotonic correlations between the selected variables.

Preliminary analysis of the survival rate was carried out with use of Cox’s Proportional Hazards Regression Analysis. Breslow’s estimate of the baseline hazard was used to compute partial likelihood in a maximum of 50 iterations. ‘Days’ is the time unit used. Models of optimal fit were proposed by means of two algoritms: backward elimination (elimination if *p* ≥ 0.15) and stepwise regression (inclusion if *p* ≤ 0.05, elimination if *p* ≥ 0.15). The models were compared in terms of fit with the use of the Akaike Information Criterion (AIC) and Schwarz Bayesian Criterion (SBC).

To illustrate the relationship between the Student's t-test results (*p* value) and fold changes in septic patients between the serum and urine samples volcano plots were made. The volcano plot shows the fold change (log2 ratio) plotted against the absolute confidence (−log10 adjusted *p* value).

The ROC (receiver operating characteristic) curves were analyzed in order to assess the potential role of selected amino acids as predictors of septic shock among septic patients. The overall performance was expressed as the area under the ROC curve (AUC). Additionally, sensitivities and specificities associated with an optimal cut-off were calculated. The optimal cut-off value was identified as the value corresponding to the highest sum of sensitivity and specificity (Youden index; J).

## Conclusions

The analysis of various metabolites that are formed during pathophysiological conditions such as sepsis or septic shock may provide a new understanding of the pathogenesis of these diseases. Moreover, this may be a helpful tool in diagnosis, prognosis or profiling therapies for sepsis or septic shock patients.

## Data Availability

The datasets generated and analyzed during the study are available from the corresponding author on reasonable request.
